# Everything is Foreseen, Yet Free will is Given (Mishna Avot 3:15)

**DOI:** 10.5334/joc.27

**Published:** 2018-05-14

**Authors:** Jeremy M. Wolfe

**Affiliations:** 1 Brigham and Women’s Hospital/Harvard Medical School, US

**Keywords:** Attention, Visual search, Visual perception

## Abstract

Jan Theeuwes’ review of visual selection shines a useful spotlight on the role of selection history in determining subsequent deployments of attention. However, he blurs an important distinction between volition and top-down guidance of attention and he underplays the role of both of those factors in the control of attention.

At the end of his interesting review, Jan Theeuwes (2018) concludes “that visual selection as conceived here … is maybe not a choice but the consequence of our experiences creating a universe that is automatically forced upon us.” I think he was closer to the mark in his title where he argued that visual selection is “Usually Fast and Automatic; Seldom Slow and Volitional”. Let us suppose that I wanted to discuss this matter with him at the opening reception of next meeting that we both attend. When I enter the meeting room, I would make a choice to search for Jan. Leaving aside philosophical issues about free will, this is a volitional act in which I somehow tell myself to perform a Theeuwes search. Thereafter, that search will run largely automatically. I will not need to make volitional choices about attending to this person and not attending to the potted plant. My attention will be deployed perhaps 20 times per second (Kwak, Dagenbach, & Egeth, 1991). It will not be strongly controlled by low-level visual salience (Henderson, Malcolm, & Schandl, 2009). There will be some role for selection history, but my recent selection history might have involved other targets like water fountains and conference center signage. I was not engaged in a block of Theeuwes search. Still, my search will not be random and that would seem to argue for something like top-down, user-driven guidance of selection. There are a couple of reasons why Theeuwes seems to give little scope to this top-down aspect of search. First, he implies that “top-down” requires moment by moment acts of volition and, second, his main interest is in tasks that don’t give top-down processes much scope.

Theeuwes makes a number of important points in this piece. As he says, volition is slow, at least when compared to automatic deployments of attention. We did a set of experiments where observers searched through the same circle of letters in two ways. In one task, they simply searched for a mirror-reversed letter. In the other task, they started at the top position and searched for the first mirror-reversed letter that could be found, moving clockwise. This required volitional deployments from item to item and was much slower than normal, “anarchic” search (Wolfe, Alvarez, & Horowitz, 2000). If you tell the observer that the mirror-reversed letter will be red, the fast, anarchic search will be guided by that top-down information. Volition is slow, but attentional selection under top-down control need not be.

Theeuwes’ emphasis on the role of selection history in search is also useful. As he notes, we had argued that priming was a form of top-down guidance (Wolfe, Butcher, Lee, & Hyle, 2003). We were making the distinction between bottom-up, stimulus-driven guidance and top-down, user-driven guidance. We put priming in the top-down category because the stimulus hadn’t changed when selection was primed by the color or shape of the previous target. But Theeuwes is right; there is something different between deliberately searching for red and having attention biased toward red by feature priming. It is not a bad idea to distinguish between effects based on the top-down intentions of the searcher and those based on the selection history and we are happy to agree that priming sensibly falls into the selection history category.

“Hybrid foraging”, a new paradigm used in our lab (Wolfe, Aizenman, Boettcher, & Cain, 2016) and in Arni Kristjansson’s (Kristjansson, Johannesson, & Thornton, 2014), can shed light on the interaction of top-down guidance and selection history processes in search. “Hybrid search” refers to simultaneous search for multiple types of targets (Schneider & Shiffrin, 1977; Wolfe, 2012). Hybrid foraging is search for multiple instances of multiple types of targets. So, let us suppose you are searching your coin bowl for American dimes (10 cent) and quarters (25 cent). There is an act of volition at the start. Thereafter, your visual search engine runs quite automatically, delivering coins to you to be picked out of the pile. If you find a quarter, the probability that the next selection will be a quarter will increase (selection history, priming). The fact that quarters have more value will increase their selection probability, too (Wolfe, Cain, & Alaoui-Soce, 2018). However, you will not collect all the quarters before selecting a dime. Your foraging will be a mix of runs of one coin and switches to the other coin. Those switches impose an RT cost (Monsell, 2003). They do not seem to require any explicit act of volition. They do seem to require some sort of top-down control, based on the task the searcher is holding in mind. Foraging behavior is not purely driven by bottom-up salience and by the history of selection. We need a role for top-down intentions, as well.

If that coin bowl contained one bright pink golf ball, that salient singleton would almost undoubtedly capture attention in a bottom-up way. However, it is possible to overstate the involuntary nature of that capture. Abrupt onsets are the classic attention capture stimulus (Jonides & Yantis, 1988). We thought we could cripple visual search by having observers look for a T among Ls while bright spots appeared at different locations every 50 msec. In fact, observers paid a very modest (50 msec) cost for the snowstorm of onsets. The onsets had no real impact on the deployments of attention in this task (Wolfe, 1996; Wolfe & Friedman-Hill, 1990).

Theeuwes’ research program has provided much of the most elegant and important work on the automatic factors that tug and pull on our attention. Sometimes, these forces act in ways that run counter to our volition. Onsets and other bottom-up factors certainly capture attention when all else is equal. However, if capture is sufficiently detrimental, it can be overruled by top-down processes. Moreover, feature singletons do not greatly disrupt the stream of searches in our life outside the lab. Nor does selection history. The item that I just searched for and found does not seem to greatly impact my search for the next object of my desires. These are components of selection, but an effective account of how we make our way in the world will require a greater appreciation of our relatively slow, volitional choices and of the fast and powerful top-down processes that those choices activate.
